# Which factor is the most effective one in metabolic Sydrome on the outcomes after coronary artery bypass graft surgery? A cohort study of 5 Years

**DOI:** 10.1186/s13019-017-0682-5

**Published:** 2018-01-04

**Authors:** Lijuan Wang, Xiangyang Qian, Mingya Wang, Xinran Tang, Hushan Ao

**Affiliations:** 1 Department of Anesthesiology, Beijing Fuwai Hospital, National Center for Cardiovascular Diseases, Chinese Academy of Medical Sciences and Peking Union Medical College, No. 167, Beilishi Road, West District of Beijing, Beijing, 100037 China; 2 Department of Cardiac Surgery, Beijing Fuwai Hospital, National Center for Cardiovascular Diseases, Chinese Academy of Medical Sciences and Peking Union Medical College, Beijing, China

**Keywords:** Metabolic syndrome, Coronary artery bypass graft surgery, Diabetes, Major adverse cerebral cardiovascular events

## Abstract

**Background:**

Metabolic Syndrome and diabetes mellitus are risk factors for cardiovascular disease. However, the effects of Metabolic Syndrome with or without diabetes on perioperative and long-term morbidity and mortality after Coronary Artery Bypass Graft remain unclear.

**Methods:**

An retrospective study was performed on 1166 patients who received isolated primary Coronary Artery Bypass Graft in Fuwai hospital. They were divided into three groups: control, Metabolic Syndrome of three factors together with diabetes and Metabolic Syndrome of three factors without diabetes (*n* = 868, 76 and 222 respectively). Analysis of variance, Chi-rank test, Fisher’s exact test, Log-rank test, Cox and Logistic regression models were used for data processing. Outcomes were postoperative and 5-year survival and morbidities.

**Results:**

There were no significant differences between groups in in-hospital postoperative complications, epinephrine use, stroke, atrial fibrillation, renal failure, coma, myocardial infarction and repeated revascularization. Patients in the Metabolic Syndrome with diabetes, Metabolic Syndrome without diabetes and control groups, respectively, showed significant difference in perioperative Major Adverse Cerebral Cardiovascular Events (30.3% vs. 21.2%, 16.7%, *P* = 0.0071) and mortality (11.8% vs. 2.7%, 3.11%, *P* = 0.0003). The Metabolic Syndrome with diabetes group had higher rates of perioperative mortality than Metabolic Syndrome without diabetes (*P* = 0.0017, P of Fisher Test = 0.0039). Compared with non-diabetic patients with Metabolic Syndrome, those with Metabolic Syndrome and diabetes had increased long-term mortality (Adjusted HR: 4.3; 95% CI: 1.4–13.3; *P* = 0.0113) and Major Adverse Cerebral Cardiovascular Events (Adjusted OR: 1.7; 95% CI: 1.0–2.8; *P* = 0.048). Control and non-diabetic Metabolic Syndrome groups did not differ in long-term mortality but controls had lower rates of Major Adverse Cerebral Cardiovascular Events (Adjusted OR: 0.79; 95% CI: 0.64–0.98; *P* = 0.0329).

**Conclusions:**

There were significance differences between the three groups in perioperative Major Adverse Cerebral Cardiovascular Events and mortality after Coronary Artery Bypass Graft. Compared with non-diabetic Metabolic Syndrome patients, patients with Metabolic Syndrome and diabetes had higher long-term Major Adverse Cerebral Cardiovascular Events and mortality. While patients free of MetS and diabetes were associated with lower incidence of long-term Major Adverse Cerebral Cardiovascular Events after Coronary Artery Bypass Graft.

## Background

The incidence of Metabolic Syndrome (MetS) has been increasing worldwide due to changing lifestyles. According to *Diagnosis and management of the metabolic syndrome: an American Heart Association; National Heart, Lung, and Blood Institute Scientific Statement*, MetS can be diagnosed as the presence of any three of the following five criteria: increased body mass index, elevated triglycerides, reduced high-density lipoprotein–cholesterol, elevated blood pressure and elevated fasting glucose [1]. The incidence of MetS in patients who receive cardiovascular surgical procedures is quite high (nearly 46%) [2] almost double the rate in the general population (23–28%) [3]. Diabetes is also increasingly more prevalent among patients with Coronary Heart Disease (CHD) who need Coronary Artery Bypass Graft Surgery (CABG). Studies show that one-quarter to one-half of MetS patients receiving CABG also suffer from diabetes [2, 4, 5]. Further, MetS is associated with higher mortality and morbidity after CABG [6, 7]. Diabetes is an independent risk factor for poor outcomes after cardiac surgery [8, 9]. One study of 235 patients found diabetes and MetS were risk factors for prolonged ICU stays (> 5 days) and atelectasia (*P* < 0.05) [10]. There was also a significant associations between diabetes and pulmonary embolism (*P* = 0.025) and mediastinitis (*P* = 0.051). However, in most studies patients with MetS may have diabetes. There are few long-term clinical studies that compared the outcomes of MetS patients with or without diabetes after CABG, especially in Chinese cohort. So it’s not clear enough about which factor in MetS contributes mostly to the bad outcomes. The present study aimed to find out the impact of MetS with or without diabetes on 30-day and 5-year mortality and MACCE in patients undergoing CABG.

## Methods

### Study design

**Fig. 1:**
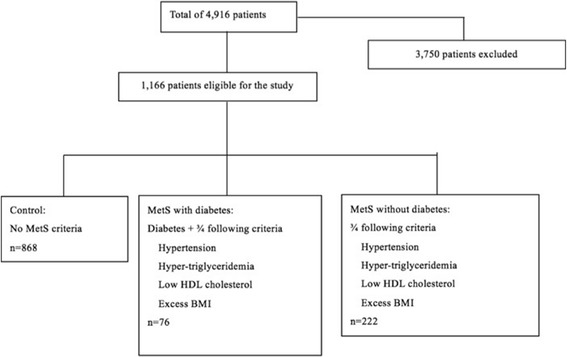
Study population recruitment summary. This study enrolled from 4916 consecutive Chinese patients who underwent isolated primary CABG at Fuwai Hospital. Among them 1166 patients met the inclusion criteria of this research, who were divided into three groups: control (*n* = 868), MetS with diabetes (*n* = 76) and non-diabetes MetS (*n* = 222)

This study enrolled from 4916 consecutive Chinese patients who underwent isolated primary CABG at Fuwai Hospital in Beijing, China. The cases were collected from January 1, 1999 to December 30, 2005. All patient records and information were anonymized and at equal prior to analysis. Authors had no access to information that could identify individual participants during or after data collection. As shown in Fig. 1, among them 1166 patients met the inclusion criteria of this research, which was sorted at 2015 December. The inclusion criteria for the patients were as follows: (1) aged more than 18 years old, (2) had a definite history of CHD (CHD was defined as having a history of myocardiac ischemia, percutaneous intervention, thrombolytic therapy or a documented angiogram with visualized luminal obstruction or irregularity), (3) no severe illness of other systems, (4) received CABG surgery, (5) no trauma, infection, tumor, or previous surgery. The 1166 patients were divided into three groups: control (*n* = 868), MetS with diabetes (*n* = 76) and non-diabetes MetS (*n* = 222). Controls were patients with no MetS criteria. The MetS with diabetes group included patients with diabetes and any 3 out of these 4 criteria: excess body mass index, hypertension, hypertriglyceridemia and low high-density lipoprotein cholesterol. The non-diabetes MetS group were patients with 3 of 4 criteria: excess body mass index, hypertension, hypertriglyceridemia and low high-density lipoprotein cholesterol with no hypertriglyceridemia or diabetes. The exact criteria of MetS can be found in the part of *Identification of patients with MetS and diabetes.* Data were obtained based on these conditions and were used to do statistical analysis.

### Operative techniques

All patients received standardized anesthetic and surgical techniques, including access through a mid-sternotomy. After surgery, patients were transferred to the ICU and extubated as soon as they were stable: body temperature at normal level, awake, hemodynamic stability and no significant bleeding.

### Identification of patients with MetS and diabetes

In this study, the clinical diagnosis of the MetS was made according to modified *Adult Treatment Panel III of the National Cholesterol Education Program* which requires meeting at least three criteria for diagnosis [11]. Obesity: body mass index greater than 28 kg/m^2^. Hyper-triglyceridemia: elevated triglycerides (>150 mg/dL or drug treatment) or low high-density lipoprotein cholesterol: reduced high-density lipoprotein cholesterol (<40 mg/dL in men, <50 mg/dL in women, or drug treatment). Hypertension: elevated arterial blood pressure (>130 mmHg systolic, >85 mmHg diastolic, or drug treatment). And hyperglycemia: elevated fasting glucose (FPG > 100 mg/dL or drug treatment). The previous criterion to classify obesity in the Adult Treatment Panel III(2005) was waist circumference > 120 cm in men, >88 cm in women, but this was not measured in this cohort. Therefore we used BMI instead of waist circumference. Recent studies showed that most MetS patients with excess BMI also have excess waist circumference [12, 13]. Diabetes mellitus (DM) was defined as a fasting plasma glucose level ≥ 126 mg/dl or requiring treatment with oral hypoglycemic medication or insulin use.

### Outcome events definition

Renal failure was defined as progressive oliguria or anuria or need for dialysis [14]. Stroke was defined as central nervous systerm (CNS) dysfunction lasting more than 72 h. Coma was defined as loss of consciousness for more than 24 h. Outcomes that ended in death and MACCE were recorded from follow-up. MACCE was defined as permanent or transient stroke, coma, perioperative myocardial infarction (MI), heart block, and cardiac arrest [15–17].

### Data sources

All these patients’ basic characteristics, perioperative data, a short-term follow-up (≤30 days after operation [18]) and a long-term follow-up (annually from the first year to the fifth year after surgery [19]) data were documented in Fuwai Hospital CABG case database. Basic characteristics and perioperative data including medical history, course in hospital, course during operation, examination and laboratory test and so on were from the electronic record of each patients. Patients’ 30 days situation and annually situation was obtained by telephone follow-up and documented in Fuwai Hospital CABG case database as well.

### Statistical analysis

Continuous variables were described in the form of mean ± standard deviation and compared by analysis of variance (ANOVA). Categorical variables were treated as frequency and percentages, then compared by Chi-square test or Fisher’s exact test. Kaplan-Meier curves represented 5-year cumulative mortalities and compared by log-rank test. Follow-up mortalities were analyzed by univariate Cox regression and multiple Cox regression models and MACCE by univariate and multiple Logistic regression models. According to previous literature, clinical observation and characteristics that showed significant difference among 3 groups in baseline variables were made to be covariates in the multiple regression models. Covariates included age, sex, smoking, aortic cross-clamp time, cerebrovascular events, peripheral artery disease, thrombolytic therapy, myocardial infarction and left main disease. All statistical tests were performed with the SAS 9.13 software (SAS Institute, Cary, NC, USA). The significance level was set at 0.05 and all tests were two-sided.

## Results

### Baseline and intraoperative characteristics

Of 4916 eligible patients in the database, 1166 patients met the inclusion criteria and were divided into three groups as shown in Fig. 1. Baseline data of the patients are presented in Table 1. No significant differences were found between the three groups in age, smoking, preoperative creatinine, cardiopulmonary bypass (CPB) time, aortic cross-clamp (ACC) time, diseased coronary artery, ejection fraction (EF) value on ultrasonic echocardiography, family history of CHD, history of peripheral vascular diseases, renal failure, left main disease, heart failure or atrial fibrillation. However, patients with MetS without diabetes tended to be female (21.2% vs. 19.7% and 11.3%, *P* = 0.0002), have stable angina pectoris (7.66 vs. 5.26% and 3.11%, *P* < 0.0001). Patients with MetS and diabetes had higher body mass index (BMI) (33 ± 24.8), were more likely to have unstable angina pectoris (22.4% vs. 15.3% and 6.80%, *P* < 0.0001), use intravenous nitrates (0.13 ± 0.34), and have a more extensive history of cerebrovascular events (7.89% vs. 5.86% and 2.53%, *P* = 0.0057). Control group members were more likely to have thrombolytic therapy (12.9% vs. 6.31%, 12.9%. *P* = 0.0050) and have a history of myocardial infarction (52% vs. 40.5%, 50.0%. *P* = 0.0099).

**Table 1:** Baseline characteristics of the patients

Variables	MetS without DM *N* = 222	MetS with DM *N* = 76	No MetS *N* = 868	*P* Value	*P* Value of Fisher Test
Age (yr)	59.97 ± 8.22	58.08 ± 7.72	58.95 ± 9.48	0.2052	
BMI (kg/m^2^)	32.89 ± 22.22	32.96 ± 24,77	24.19 ± 2.26	<0.0001	
Smoking	131 (59.01%)	47 (61.84%)	489 (56.34%)	0.5406	
Creatinine (tjmol/L)	103.42 ± 110.68	133.75 ± 196.34	110.38 ± 123.25	0.5821	
CPU time (min)	58.88 ± 66.51	63.88 ± 64.48	58.48 ± 76.78	0.8309	
ACC Time (min)	36.89 ± 42.16	41.37 ± 42.53	36.39 ± 40.29	0.5945	
Diseased coronary artery	2.77 ± 0.50	2.72 ± 0.48	2.7110.57	0.2480	
LVEF	59.73 + 9.34	58.41 + 8.36	59.15 + 10.04	0.5599	
Preoperative intravenous use of nitrates	0.06 ± 0.24	0.13 ± 0.34	0.0510.21	0.0096	
Female	47 (21.17%)	15 (19.74%)	98(11.29%)	0.0002	
Hypertension	222 (100%)	76 (100%)	0	<0.0001	
Hyperlipidemia	222 (100%)	76 (100%)	0	<0.0001	
Cerebrovascular events	13 (5.86%)	6(7.89%)	22 (2.53%)	0.0057	
Renal failure	0	0	2 (0.23%)	0.7090	1.0000
Elevated fasting glucose	0	76(100%)	0	<0.0001	
Thrombolytic therapy	14 (6.31%)	4(5.26%)	112 (12.90%)	0.0050	
PCI history	0	0	0		
Angina pectoris					
Unstable angina pectoris	34 (15.32%)	17 (22.37%)	59 (6.80%)	<0.0001	
Stable angina pectoris	17 (7.66%)	4(5.26%)	27(3.11%)	<0.0001	
Myocardial infarction	90 (40.54%)	38 (50.00%)	451 (51.96%)	0.0099	
Family history	21 (9.46%)	5 (6.58%)	54 (6.22%)	0.2333	
Left main disease	55 (24.77%)	20 (26.32%)	270 (31.11%)	0.1481	
Peripheral artery disease	21 (9.46%)	4(5.26%)	63 (7.26%)	0.3994	
Heart failure	2 (0.90%)	2 (2.63%)	18 (2.07%)	0.4590	0.4242
Atrial fibrillation	6 (2.70%)	3 (3.95%)	15 (1.73%)	0.3211	0.2247

### Perioperative (short-term) outcomes

Table 2 helps illustrate that there were no significant differences in ICU stay, ventilation time, in-hospital postoperative complications, epinephrine use, stroke, atrial fibrillation, renal failure, coma, myocardial infarction and repeated revascularization among the groups. The groups did differ significantly in perioperative MACCE (30.3% vs. 21.2% and 16.7%, *P* = 0.0071) and mortality (11.8% vs. 2.7% and 3.11%, *P* = 0.0003). As the Chi-square and Fisher’s Exact test results in Table 3 show, the MetS with diabetes group had higher rates of perioperative mortality than Mets without diabetes (*P* = 0.0017, P of Fisher Test = 0.0039), while rates of MACCE were equal. No significant differences in mortality or MACCE were found between the Mets without diabetes and control groups.

**Table 2:** Postoperative characteristics

Variables	MetS without DM *N* = 222	MetS with DM *N* = 76	No MetS *N* = 868	*P* Value	*P* Value of Fisher Test
ICU stay(h)	60.33 ± 67.79	48.26 ± 38.29	61.06 ± 63.92	0.2400	
Ventilation Time (h)	18.23 ± 22.63	16.22 ± 7.12	15.86 ± 24.17	0.3938	
Postoperative Complications	0	0	7 (0.81%)	0.2985	0.5986
Epinephrine Use	28 (12.61%)	14 (18.42%)	93 (10.71%)	0.1141	
Stroke	1 (0.45%)	1 (1.32%)	4 (0.46%)	0.6005	0.4018
Atrial Fibrillation	13 (5.86%)	4 (5.26%)	62 (7.14%)	0.6846	
Renal Failure	1 (0.45%)	1 (1.32%)	1 (0.12%)	0.1151	0.0815
Coma	2 (0.90%)	0	7 (0.81%)	0.7214	1.0000
Myocardial Infarction	3 (1.35%)	2 (2.63%)	8 (0.92%)	0.3693	0.2403
Repeated Revascularization	5 (2.25%)	3 (3.95%)	33 (3.80%)	0.5232	
Mortality	6 (2.70%)	9 (11.84%)	27 (3.11%)	0.0003	
MACCE	47 (21.17%)	23 (30.26%)	145 (16.71%)	0.0071

**Table 3:** MetS and Perioperative (Short-term) Mortality and MACCE

Variables	Mortality		MACCE	
	*P* Value	*P* Value of Fisher Test	*P* Value	*P* Value of Fisher Test
MetS with DM vs. MetS without DM	0.0017	0.0039	0.1066	0.1181
No MetS vs. MetS without DM	0.1002	1.0000	0.1190	0.1382
MetS with DM vs. No MetS	0.0001	0.0014	0.0030	0.0048

### Long-term follow-up for mortality

**Fig. 2:**
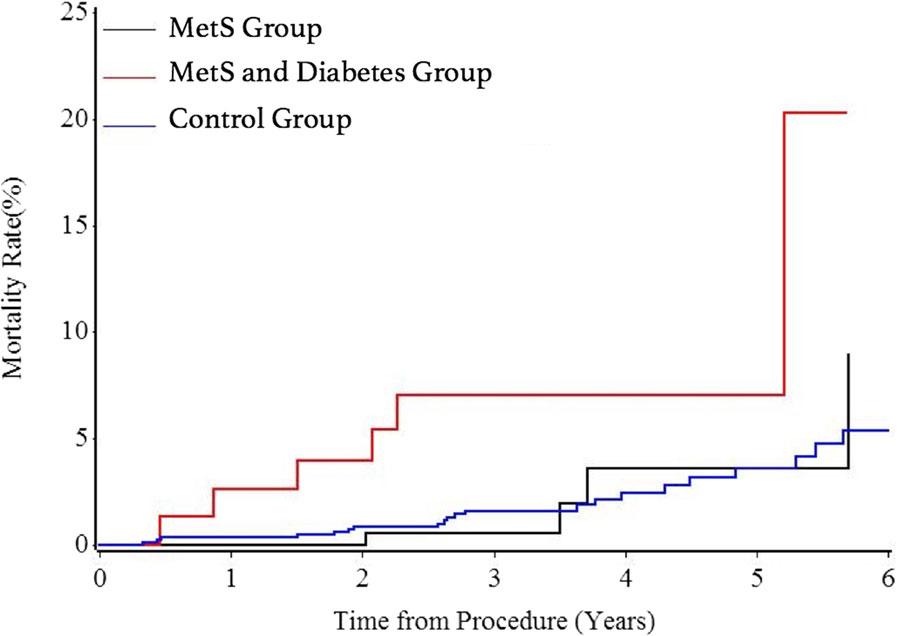
Kaplan-meier for mortality rate following-up 5 years. MetS with diabetes group showed significant higher mortality than control group during the annual follow-up while Mets without diabetes group showed no significant difference. (Log-rank test *P* = 0.0075)

The median follow up duration was 59.3 months. Compared to non-diabetetic MetS group, patients with MetS and diabetes suffered significantly greater long-term mortality (HR: 3.048; 95%CI: 1.022–9.086; *P* = 0.0456). There was no significant difference between the non-diabetic MetS and control groups (Table 4).The Kaplan-Meier curves in Fig. 2 illustrate the trend of long-term mortality among three groups. MetS with diabetes group showed significant higher mortality than control group during the annual follow-up while Mets without diabetes group showed no significant difference. (Log-rank test *P* = 0.0075). Multivariable Cox regression model was used to adjust the confounding factors and analyze the association among MetS, diabetes and mortality. As shown in Table 5, patients in MetS with diabetes group had increased long-term follow-up mortality (adjusted HR: 4.299; 95% CI: 1.392–13.277; *P* = 0.0113). In addition, old age (adjusted HR: 1.061; 95% CI: 1.021–1.103; *P* = 0.0027) and smoking (adjusted HR: 2.103; 95%CI: 1.019–4.341; *P* = 0.0443) were also risk factors for the long-term mortality.

**Table 4:** MetS and Long-term Mortality

Variables	Un-adjusted				Adjusted	
	HR	95% Cl of HR	*P* Value	HR	95% CI of HR	*P* Value
MetS with DM vs. MetS without DMs	3.048	1.022–9.086	0.0456	4.299	1.392–13.277	0.0113
No MetS vs. MetS without DM	0.862	0.354–2.096	0.7426	0.992	0.388–2.540	0.9870

**Table 5:** Multivariable-adjusted hazard ratio of long-term mortality

Variables	*P*-Value	HR	HR9S% CI
MetS with DM	0.0113	4.299	1.392–13.277
Non MetS	0.9870	0.992	0.388–2.540
Age	0.0027	1.061	1.021–1.103
Aortic Cross-clamp Time	0.3390	0.996	0.988–1.004
Sex	0.8026	0.867	0.282–2.660
Smoking	0.0443	2.103	1.019–4.341
Cerebrovascular Events	0.2245	2.143	0.626–7.331
Peripheral Artery Disease	0.0904	2.537	0.864–7.450
Thrombolytic Therapy	0.3519	1.535	0.623–3.787
Myocardial Infarction	0.5509	0.812	0.410–1.609
Left Main Disease	0.9144	1.038	0.528–2.040

### Long-term follow-up for MACCE

The univariate Logistic regression model showed increse in long-term MACCE in the MetS with diabetes compared to non-diabetes MetS groups (Table 6). Patients in the Control group had a lower incidence of MACCE compared to the non-diabetes MetS group (OR: 0.741; 95% CI: 0.601–0.913; *P* = 0.0050). Multivariable Logistic regression adjusted the confounding factors to analyze long-term MACCE. As in Table 7, patients in MetS with diabetes group showed increasing rates of MACCE (adjusted OR: 1.674; 95% CI: 1.004–2.792; *P* = 0.0484). Patients in control group had decreasing rate of MACCE (Adjusted OR: 0.792; 95% CI: 0.639–0.981; *P* = 0.0329). In addition, old age (Adjusted OR: 1.029; 95% CI: 1.017–1.041; *P* < 0.0001), cerebrovascular events (Adjusted OR: 1.964; 95% CI: 1.395–2.767; *P* = 0.0001) and aortic cross-clamp time (Adjusted OR: 1.005; 95% CI: 1.003–1.007; *P* < 0.0001) are also risk factors for the rate of MACCE.

**Table 6:** MetS and MACCE in the 5-year follow-up

Variables	Un-adjusted				Adjusted	
	OR	95% CI of OR	*P* Value	OR	95% CI of OR	*P* Value
MetS with DM vs. MetS without DM	1.603	0.971–2.647	0.0652	1.674	1.004–2.792	0.0484
No MetS vs. MetS without DM	0.741	0.601–0.913	0.0050	0.792	0.639–0.981	0.0329

**Table 7:** Multivariable-Adjusted Odd Ratio of Long-term MACCE

Variables	*P*-Value	OR	OR 95% CI
MetS with DM	0.0484	1.674	1.004–2.792
Non MetS	0.0329	0.792	0.639–0.981
Age	<.0001	1.029	1.017–1.041
Aortic Cross-clamp Time	<.0001	1.005	1.003–1.007
Sex	0.8247	0.969	0.732–1.283
Smoking	0.0704	1.207	0.984–1.480
Cerebrovascular Events	0.0001	1.964	1.395–2.767
Peripheral Artery Disease	0.9246	0.983	0.696–1.389
Thrombolytic Therapy	0.1203	1.286	0.936–1.766
Myocardial Infarction	0.3447	1.100	0.903–1.339
Left Main Disease	0.1434	0.857	0.697–1.054

## Discussion

In the present study, differences of both short and long term mortality were found between diabetic MetS patients and non-diabetic MetS patients, while were not found between non-diabetic MetS patients and non-MetS paients, which indicated that diabetes played an important role in the death of MetS patients. We also found that, compared with non-diabetic MetS patients, diabetic MetS patients had higher rates of MACCE, and so did non-diabetic MetS patients when compared with control patients. So both MetS and diabetes may contributes to the development of MACCE. Besides, age, smoking, cerebralvascular events and aortic cross-clamp time showed in the substudies were also the risk factors of mortality or morbidity after CABG.

There’s close contact between atherosclerosis, Metabolic Syndrome and diabetes. A conception called “Cardiometabolic Diseases” revolve around a complicated cluster of events including visceral adiposity, MetS, type 2 diabetes and CHD [20]. Insulin resistance is the major contributors to the pathological basis of MetS and Diabetes (which may contains confounding factors). Glucolipotoxicity and chronic inflammatory state generated under insulin resistance lead to dysfunction of VEC. On the other hand, pancreatic dysfunction from islet VEC leads to low perfusion of pancreas islet and aggravates the decompensation. In addition, one study has shown that diabetes tends to leave patients in a hypercoagulable state [21]. These abnormalities work together in developing atherosclerosis. Complex coronary lesions, such as bifurcation and ostial lesions, are significantly more common in diabetic patients [22].

In cardiovascular surgery, CPB, hypothermic anesthesia and blood dilution trigger intense stress reactions, which are characterized by hyperglycemia and hyperinsulinemia. And the ischemia-reperfusion injury of myocardium reduces the reaction of myocardial cells to insulin, i.e., myocardial insulin resistance. A further study found that patients with blood glucose values >200 mg/dl immediately after CABG had an increased risk of complications, including mortality [9]. Another study reported chronic hyperglycemia is associated with acute kidney injury after CABG [23].

MetS and diabetes are becoming increasingly common as the result of worldwide change in diet and lack of exercise, leading to enormous economic burdens on society [24–26]. CVD already is the most common cause of death in China. Determining the impacts of MetS and diabetes on outcomes after CABG will help to manage operative risk for a growing proportion of the population. Furthermore, it is crucial for the development of public health policy and clinical guidelines for prevention and treatment which may lead to a controlled form of MetS and diabetes, potentially improving long-term outcomes after CABG.

But very few studies have previously attempted to delineate the role of MetS and/or diabetes on the immediate and long-term MACCE and mortality. This study has filled in the blank to some extent.

Studies have found contradictory results about the effect of MetS on outcomes after CABG. In the mainstream view, MetS predicts outcomes after CABG [4, 6, 7, 10]. Recently contributors propose that MetS has no detrimental effect on either the pre-operative risk factors or the outcome after CABG [27, 28]. Further experiments are necessary to solve the mystery.

Contradictory results exist on the effect of diabetes on outcomes after CABG. Combining 146,000 patients from various hospitals showed a mortality of 3.7% for patients with diabetes mellitus and 2.7% for those without diabetes mellitus [29]. A propensity-matched study containing 1122 subjects showed that long-term survival is significantly lower in patients with diabetes compared to non-diabetics after CABG [30]. A survival analysis of 910 CABG patients by the Life Table method found that patients without diabetes had at least equal survival in 16 years after CABG compared to their matched background populations. While survival of DM patients started to decline few years after the operation [31]. But in these studies, DM or non-DM suffered the influence of MetS, which may show more strongly in DM group.

There are also researches involvles both MetS and diabetes attempting to find out their influnces on the outcomes of CABG [32]. In a 15-year observational study MetS increased all-cause and cardiac mortality of non-diabetic patients (hazard ratio 1.34, *P* = 0.028 and 2.31, *P* = 0.002, respectively) while no increased mortality was found among diabetic patients [4]. The result of a retrospective reseach showed the motality of diabetic patients with no MetS after CABG had no difference with patients with neither diabetes nor MetS, however, patients with both diabetes and MetS were found out worse than diabetic patients without MetS which conflict with the former [2]. And in the same research, the risk of mortality was increased by 2.69-fold (95% CI 1.43 to 5.06; *p* = 0.002) in patients with MS and diabetes and 2.36-fold (95% CI 1.26 to 4.41; *p* = 0.007) in patients with MS and no diabetes compared with no diabetes and no MetS patients while there’s no comparison between the two cohort. In this study, we found that patients with MetS plus diabetes had higher rates of postoperative and long-term mortality and MACCE compared with MetS patients without diabetes. However, patients with diabetes and other 2 criteria of MetS might be included in MetS with diabetes in the two researches mentioned above but excluded in current study. Besides, difference of the diagnostic criteria of MetS and statistical methods adapted in different articles may also have influences to the conclusion. In the future, it’s of meaning to take non-diabetic 3- factors MetS, non-MetS diabetes and diabetes plus 3-factors MetS or non-diabetic 3- factors MetS and 2- factors MetS plus diabetes into comparison to find out the further role of MetS or diabetes in the outcome of CABG. Based on which, a risk stratification used to conduct preoperative preparation or even the whole perioperative management maybe possible.

### Limitations

This was a retrospective study at a single center among a cohort of exclusively Chinese patients. Bias may remain despite multivariate adjustments to reduce overt sources. Also, MetS was assessed using BMI because waist circumferences were not available. Although international guidelines suggest the use of waist circumference to classify obesity, several studies have demonstrated that there is no significant difference between the two classification methods, i.e., BMI or waist circumference.

## Conclusions

There were significance differences between the three groups in perioperative Major Adverse Cerebral Cardiovascular Events and mortality after Coronary Artery Bypass Graft. Compared with non-diabetic Metabolic Syndrome patients, patients with Metabolic Syndrome and diabetes had higher long-term Major Adverse Cerebral Cardiovascular Events and mortality. While patients free of MetS and diabetes were associated with lower incidence of long-term Major Adverse Cerebral Cardiovascular Events after Coronary Artery Bypass Graft.

## Abbreviations

AGE: Advanced glycosylation end products
BMI: Body mass index
CABG: Coronary artery bypass graft
CHD: Coronary heart disease
CNS: Central nervous system
CPB: Cardiopulmonary bypass
DM: Diabetes mellitus
EF: Ejection fraction
MACCE: Major adverse cerebral cardiovascular events
MetS: Metabolic syndrome
MI: Myocardial infarction
NIDDM: Non insulin dependent diabetes mellitus
VEC: Vascular endothelial cells
